# The Chemophytostabilisation Process of Heavy Metal Polluted Soil

**DOI:** 10.1371/journal.pone.0129538

**Published:** 2015-06-26

**Authors:** Anna Grobelak, Anna Napora

**Affiliations:** Institute of Environmental Engineering, Czestochowa University of Technology, 42200, Czestochowa, Poland; Friedrich Schiller University, GERMANY

## Abstract

Industrial areas are characterised by soil degradation processes that are related primarily to the deposition of heavy metals. Areas contaminated with metals are a serious source of risk due to secondary pollutant emissions and metal leaching and migration in the soil profile and into the groundwater. Consequently, the optimal solution for these areas is to apply methods of remediation that create conditions for the restoration of plant cover and ensure the protection of groundwater against pollution. Remediation activities that are applied to large-scale areas contaminated with heavy metals should mainly focus on decreasing the degree of metal mobility in the soil profile and metal bioavailability to levels that are not phytotoxic. Chemophytostabilisation is a process in which soil amendments and plants are used to immobilise metals. The main objective of this research was to investigate the effects of different doses of organic amendments (after aerobic sewage sludge digestion in the food industry) and inorganic amendments (lime, superphosphate, and potassium phosphate) on changes in the metals fractions in soils contaminated with Cd, Pb and Zn during phytostabilisation. In this study, the contaminated soil was amended with sewage sludge and inorganic amendments and seeded with grass (tall fescue) to increase the degree of immobilisation of the studied metals. The contaminated soil was collected from the area surrounding a zinc smelter in the Silesia region of Poland (pH 5.5, Cd 12 mg kg^-1^, Pb 1100 mg kg^-1^, Zn 700 mg kg^-1^). A plant growth experiment was conducted in a growth chamber for 5 months. Before and after plant growth, soil subsamples were subjected to chemical and physical analyses. To determine the fractions of the elements, a sequential extraction method was used according to Zeien and Brümmer. Research confirmed that the most important impacts on the Zn, Cd and Pb fractions included the combined application of sewage sludge from the food industry and the addition of lime and potassium phosphate. Certain doses of inorganic additives decreased the easily exchangeable fraction from 50% to 1%. The addition of sewage sludge caused a decrease in fraction I for Cd and Pb. In combination with the use of inorganic additives, a mobile fraction was not detected and an easily mobilisable fraction was reduced by half. For certain combinations of metals, the concentrations were detected up to a few percent. The application of sewage sludge resulted in a slight decrease in a mobile (water soluble and easily exchangeable metals) fraction of Zn, but when inorganic additives were applied, this fraction was not detected. The highest degree of immobilisation of the tested heavy metals relative to the control was achieved when using both sewage sludge and inorganic additives at an experimentally determined dose. The sequential extraction results confirmed this result. In addition, the results proved that the use of the phytostabilisation process on contaminated soils should be supported.

## Introduction

Heavily industrialised and urbanised areas of soil degradation are primarily related to the deposition of heavy metals (HM). These pollutants mainly result from mining activities and from processing non-ferrous metals and energy. In addition, HMs represent a very specific group of contaminants that are not biodegradable and cannot decompose into simple compounds. Thus, HMs accumulate in the food chain and result in toxic effects for live organisms. In addition, areas contaminated with heavy metals pose serious risks associated with secondary emissions sources. For example secondary emissions can result from increased wind across surfaces with no cover crops and can result in the leaching and migration of through the soil profile and to the groundwater [1, 2, 3].Thus, in these areas, the optimal solution is the application of remediation methods that enable the creation of conditions for restoring plant cover and ensure that the groundwater is protected against pollution.

The activities applied to large-scale remediation of surfaces contaminated with metals should mainly focus on limiting the degree of metal mobility in the soil profile and on decreasing their bioavailability to levels below their phytotoxic levels. Therefore, the remediation of such sites using minimally invasive methods, such as assisted phytostabilisation, is reasonable. Soil amendments allow plants to grow and can increase their metal immobilisation abilities. Some methods based on the application of chelating agents has shown an opposite effect, in increasing the solubility of heavy metals [4]. In addition, the phytostabilisation method is applied for the physical stabilisation of soils to minimise contaminant migration and bioavailability and to increase the precipitation of contaminants as insoluble salts. Furthermore, this method of using appropriately selected plant species can improve the soil parameters and affect the preferred fertilisation method [5]. Chemophytostabilisation is a complex process that is affected by numerous chemical, physical and biological reactions.

The effectiveness of phytoremediation methods depend on many factors, mainly on the bioavailability of pollutants to the roots of the plants and the processes of absorption, translocation and accumulation of contaminants by plants. The bioavailability of metals plays an important role in both approaches. Recent studies have shown that the transformation of pollutants in the soil is a dynamic process and that the bioavailability of metals varies with time [6]. Phytostabilisation involves the use of plants and soil additives to stabilise the physical and chemical immobilisation of soil contaminants [7, 8]. Phytostabilisation does not physically remove contaminants from the soil, but causes their deactivation and immobilisation (biologically unavailable form).

Chemophytostabilisation is a process in which soil amendments are distributed in and/or mixed in the topsoil [9]. The bioavailability of metals (and metalloids) in the soil environment is defined as a fraction of the total metal content in the soil solution and the soil particles that are available to the receptor [10]. Organic additives affect contaminant concentrations, including heavy metal concentrations, through a number of processes, such as immobilisation, reduction, evaporation or modification of the plant rhizosphere [9]. Increasing the retention of metals in the soil results from increased surface electric charges and the presence of metal binding particles [11]. This increase occurs following compost addition, which increases the surface charge [12]. In addition, compost can be used as a biosorbent for the removal of acid dyes from wastewater that is generated by industry [13, 14]. The presence of phosphate ions, Al, Fe, Mn compounds and other inorganic minerals in municipal sewage sludge is considered to increase the sorption capacity of organically fertilised soils [15]. The application of organic additives to the soil has the highest impact on the rhizosphere zone. After the application of organic additives, numerous changes are observed in the chemical (e.g., pH, organic acids, the composition of the soil solution) and biological (e.g., microbial) soil properties [16]. Chemical changes in the soil, which result from the addition of organic matter and plant growth, can affect the transformation, mobility and bioavailability of metals [17]. The use of organic additives, such as fertilisers and animal biomaterial, often lowers the soil pH in the rhizosphere [18]. One solution to the problem of organic matter mineralization is the simultaneous use of inorganic additives that affect the immobilisation of heavy metals and increase the soil pH. The overall result would provide a long-term effect of heavy metal immobilisation in the soil. Thus, this method could be used for degraded areas with compact vegetation cover.

Neutralisation of acidic or alkaline soils is one of the simplest and least expensive methods for immobilising heavy metals in contaminated soils [19]. Phosphate minerals can absorb and/or precipitate metals. The sorption of metals (Cd, Cu, Ni, Pb and Zn) on the surface of the hydroxyapatite indicates that the formation of complexes on the surface of hydroxyapatite and coprecipitation are the most important mechanisms for the immobilisation of Zn and Cd. Apatite was more effective than lime for reducing the Zn concentrations in plant leaves, especially when the soil contained high concentrations of Zn [20].

The phytostabilisation technique is useful for immobilising many heavy metals including, for example, Pb, As, Cd, Cr, Cu, and Zn [21, 22, 23, 24]. In addition, phytostabilisation can be used to restore vegetation cover in areas with limited vegetation due to high levels of pollution. In this case, phytostabilisation is the preferred process limiting the migration of pollutants in the environment by reducing the secondary dusting and water erosion. In addition, the same method is very effective when contamination must be immobilised quickly to protect ground and surface waters [25]. Devastated areas require reclamation, which must begin with the initiation of soil forming processes and vegetation restoration [26]. During the development of vegetation cover, the soil physical and chemical parameters (pH, carbon content, particle size) in a portion of the substrate improve, indicating that soil-forming processes occur in in areas with vegetation cover. The reclamation of degraded soils and soil deprived of a functional layer generally requires the use of pioneering plants, such as grass, which are capable of growing in extreme conditions lacking top soil or in soils with high metal contents [21, 26].

The main objective of this study is to investigate the effects of soil organic and inorganic amendments on the assisted phytostabilisation process of Cd, Pb and Zn conducted with fescue grass. This investigation includes the identification of contaminant forms and the examination of heavy metal bioavailability to determine the effects of additives on the migration of metals in the soil and into the biomass of plants. The practical aim of this research is to limit the migration of micropollutants (metals) into the groundwater in large areas that are contaminated with heavy metals (HM). The direct aim of this work is to study the solubility and solid phase fractionation of metals in podzolic soils from the metallurgical activity area in Miasteczko Slaskie in the Silesia region (Poland).

## Materials and Methods

### Ethics Statement

This study was carried out on collectively owned lands with the permission of the owners. The field studies did not involve endangered or protected species.

### Sampling site and materials characterisation

Contaminated soils were collected from a direct exposure area of a zinc smelter plant in Miasteczko Slaskie, in the Silesia region of Poland (GPS 50.510397, 18.936439). Nearly the entire region consists of Quaternary sediments in the form of boulder clay and sand clay that underlie very poor soils, sand, gravel, aeolian sand and dunes. The groundwater table is discontinuous in the Quaternary, sandy soils and varies from 1.0 to 4.0 m. The climate is a typical temperate climate with an average rainfall of 700–780 mm year^-1^ during the summer. The studied zinc smelter plant has been in operation since 1969 and is one of the main producers of Zn, Pb and Cd in Poland. According to the Central Statistical Office, the annual Zn production is 85,000 Mg, which accounts for approximately 40% of the total domestic production and approximately 50% of the total Pb production.

The soils in the study area were contaminated with metals, especially Cd, Pb and Zn, moreover, the soils of the study area were also acidified (Table 1). The soil used in the experiment is classified as a sandy soil with 2.2% gravel, 96% sand, and 1.8% clay. The natural vegetation is very poor and consists of thickets containing xerophitic species and deformed pinus trees.

**Table 1:** Selected chemical and physical characteristics of the contaminated soil and sewage sludge.

Parameter	Soil ±SD	Sewage sludge ±SD
**Humidity [%]**	18.46±0.97	90 ±0.43
**CEC [cmol(+) kg** ^**-1**^ **d.m.]**	3.60±0.41	Nd
**pH in H**_**2**_ **O**	5.3±0.4	7.01
**pH in 1 M KCl**	4.80±0.63	6.85
**Humic acid %**	0.80±0.04	Nd
**Total C [g kg** ^**-1**^ **d.m.]**	14.30 ±1.1	314±23.87
**Total organic C [g kg** ^**-1**^ **d.m.]**	13.21±3.19	300.2±14.76
**Kjeldhal N [g kg** ^**-1**^ **d.m.]**	0.95±21.78	15±2.65
**Available P [mg kg** ^**-1**^ **d.m.]**	18.97±4.76	2461.35±54.17
**Total P [mg kg** ^**-1**^ **d.m.]**	71±5.1	3479.73±61.23
**Total heavy metal concentrations [mg kg** ^**-1**^ **DM]**		
**Cd**	15.8±0.5	1.2±0.8
**Zn**	773±7.63	270±18
**Pb**	1290.7±15.4	130±9
**Bioavailable (CaCl**_**2**_ **extraction) heavy metal concentrations [mg kg** ^**-1**^ **DM]**		
**Cd**	0.98±0.07	0.2±0.01
**Zn**	11±0.06	80±5
**Pb**	39.7±1.25	12±0.7

In this research, several soil amendments were used, including sewage sludge and inorganic additives. In these experiments, aerobically stabilised sludge was used from a plant producing mineral water and juices. This sewage sludge can be used for soil reclamation due to its characteristics and low metal contents (Table 1). The organic biosolid used in this study (% m/m addition) consisted of anaerobically digested sewage sludge (Table 2) from the food industry. Potassium phosphate (PP), a finely crystalline, completely water-soluble fertiliser consisting of 52% P_2_O_5_ and 34% K_2_O, was used. In addition, granular triple superphosphate (SP), consisting of a mixture of calcium hydrogen phosphate and gypsum with 46% P_2_O_5_, was used. Finally, agricultural mixed lime (AML), as a mixture of ground limestone (CaCO_3_) and quicklime consisting of 50% CaO, was used. The addition of sand was used for exclusion the dilution effect.

**Table 2:** The applied soil amendments.

Variant abbreviation	Amendments composition
K	control
K1	sand 1% + AML 0.5%
K2	sand 3% + 0.5% AML
K3	1% sewage sludge +0.5% AML
K4	3% sewage sludge +0.5% AML
K5	0.5% AML + 0.8% SP
K6	0.5% AML + SP 0.8% + 1% sewage sludge
K7	0.5% AML + SP 0.8% +3% sewage sludge
K8	0.5% AML + PP 0.8%
K9	0.5% AML + PP 0.8% +1% sewage sludge
K10	0.5% AML + PP 0.8% +3% sewage sludge

potassium phosphate (PP), granular triple superphosphate (SP), agricultural mixed lime (AML), % m/m

### Experimental procedure

The contaminated topsoil used in this study was collected in the spring. A pot experiment was performed by applying sewage sludge and inorganic amendments, including potassium phosphate (PP), superphosphate (SP), and agricultural mixed lime (AML). Soil samples were obtained from the top of the soil (30 cm deep) and were reweighed and mixed with sewage sludge (accounting for 1% and 2% d.w.) and inorganic amendments at 0.8% each. For variants of the soil and the soil with sewage sludge, liming was conducted at 0.5%. This addition of lime was used to increase the pH to 7. The prepared samples were transferred to pots that could hold 1 kg of the soil material. In addition, control pots without any amendment were prepared using three replicates for each combination. Subsequently, pots were left for a two-week incubation period in phytotron (pots were irrigated twice a week to maintain a constant moisture content and prevent dryness), and then the fescue grass (FE) (*Festuca arundinacea* Schreb.) was seeded in an amount of 4 g of grass seeds per each pot (φ 15 cm) at a depth of 0.5 cm and irrigated daily. Pots without vegetation and with and without the amendments were used as controls.

A plant growth was conducted in a phytotron for 4 months under artificial light (350 μmol m^-2^ s^-1^) and temperature a temperature of 20°C during the day and 14°C during the night at 70% relative humidity. During the plant growth only the irrigation with deionised water was applied as needed (15 mL every two days for each pot). The plants growth lasted for 4 months. After that period biomass was harvested and roots were rinsed under running water and next in deionised water. Roots and shoots samples were dried at 70°C for 48 h and then were ground. Soil samples were obtained from each pot from the plant rhizosphere. The root system, together with the adhering soil, was carefully removed from the pots. The rhizosphere soil that adhered to the roots was carefully collected by gently shaking the root system.

### Analytical methods

The biosolids were dried and ground prior to analysis and a series of analyses were conducted prior to characterisation. Before and after the plant growth experiment, soil subsamples were air-dried and passed through a 2 mm mesh screen. The following parameters were determined for the subsamples as described in detail previously [27]: pH (in H_2_O and KCl deionised water suspension using a soil to water ratio of 1:2.5) (PN-ISO 10390:1997), CEC (Cation Exchange Capacity) according to Kappen’s method, humic acids contents, total Kjeldahl N (PN-ISO 11261:2002), total organic carbon using a Multi N/C H1300 Analitykjena (PN-ISO 10694:2002), available P using the Enger-Riehm method, total P (PN-ISO 11263:2002), and the soil moisture contents (PN-ISO 11465:1999). The soil solution samples were filtered through a Whatman 0.45 μm filter prior to pH analysis. The plant materials (shoots and roots) were weighted separately (0.3 g), and the sewage sludge (0.3 g) and soil (0.5 g) materials were digested in triplicate using hot aqua regia (PN-ISO 11047:2001) (1995), and then subjected to analysis using inductively coupled plasma optical emission spectrometry (ICP-OES; Thermo apparatus). For analysis the reference materials were taken into account: plant material (ERM-CD281), soil material (LGCQC3004), and sewage sludge material (BCR-146R). For the sequential extraction, the procedure of Zeien and Brummer (1989) [28] was followed. All suspensions were centrifuged at 2500 rpm for 10 min and passed through a 0.45 μm filter and then ICP analysed. Briefly, the seven metals fractions were extracted (Table 3).

**Table 3:** Element fractionation according to Zeien and Brummer (1989).

Fraction	Name of the fraction	Extracting compounds	Extraction time h	pH
**I**	Mobile (water soluble and easily exchangeable metals)	1 M NH_4_NO_3_	24	Natural
**II**	Easily mobilisable (specifically adsorbed, bound to CaCO3)	1 M CH_3_COONH_4_・	24	6.0
**III**	Bound to Mn oxides	1 M NH_2_OH・HCl・+ 1 M CH_3_COONH_4_・	0.5	6.0
**IV**	Bound to organic matter	0.025 M C_10_H_22_N_4_O_8_・	1.5	4.6
**V**	Bound to amorphous Fe oxides	0.2 M (NH_4_)_2_C_2_O_4_・+ 0.2 M H_2_C_2_O_4_・	4	3.25
**VI**	Bound to crystalline Fe oxides	0.2 M (NH^4^)_2_C_2_O_4_・+ 0.2 M H_2_C_2_O_4_・+ 0.1 M C_6_H_8_O_6_	0.5	3.25
**VII**	Residual fraction	using *aqua regia* (HNO3:HCl, 1:3 v/v)	-	-

### Statistical treatment of data

All statistical analyses, as described in detail previously [27], were conducted using the Statistica 6.0 software (StatSoft, Inc., 2001). The one-way analysis of variance (ANOVA) was applied, and the data not satisfying assumptions for ANOVA were analysed using the Kruskal–Wallis non-parametric test. If significant differences were observed (p<0.05), a post hoc Tukey’s honest significant difference (HSD) test was used to further elucidate differences among the means (p<0.05). Moreover, between the total and bioavailable concentrations of heavy metals in the soil and shoots and the relative plant growth, the Pearson’s correlation coefficients were calculated.

## Results

### Changes in the heavy metals contents in the soil material and the resulting biomass

**Fig 1:**
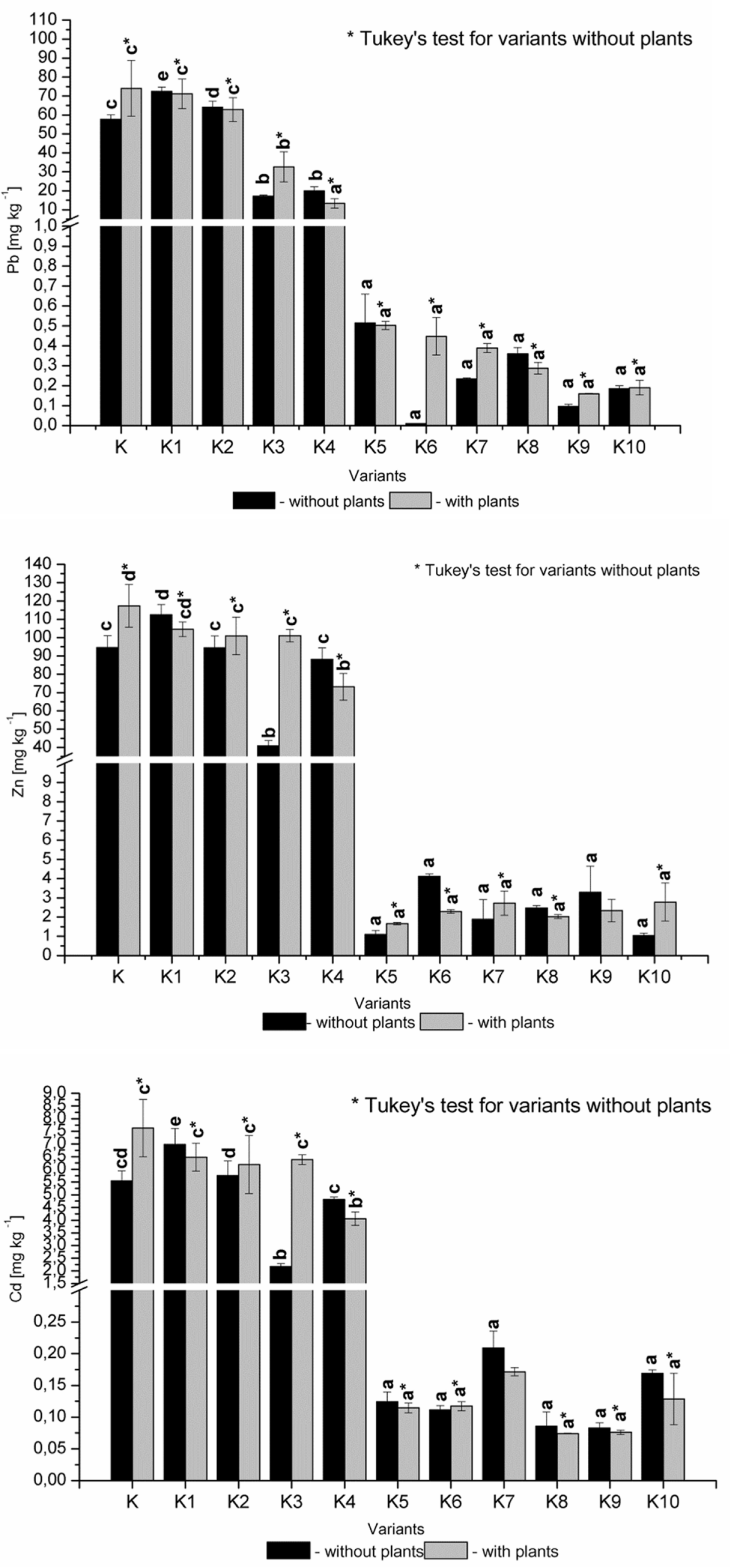
Soil bioavailable concentrations of Pb, Zn and Cd [mg kg ^-1^ DW] in the soil after different treatments.

In this experiment the soil liming treatment was conducted to maintain a constant pH after 4 months of the experiment (6–6.5). The K5-K10 variants significantly decreased the bioavailable forms of trace elements after extraction with 0.01 M CaCl_2_ (Fig 1). In this case, Cd decreased from 7.5–2.5 to 0.2–0.1 mg kg^-1^, Pb decreased from 70–30 to 0.5–0.01 mg kg^-1^ and Zn value decreased from 80–120 to 1–4 mg kg^-1^. The introduction of sewage sludge (K3, K4), solely resulted in a slight decrease in the bioavailable forms of Cd and Zn. This amendment decreased by half bioavailable forms of Pb. In addition, the best results were obtained after the introduction of inorganic additives, where the Cd and Pb bioavailable concentrations were less than 1 mg kg^-1^, and the Zn concentration was less than 5 mg kg^-1^.

**Fig 2:**
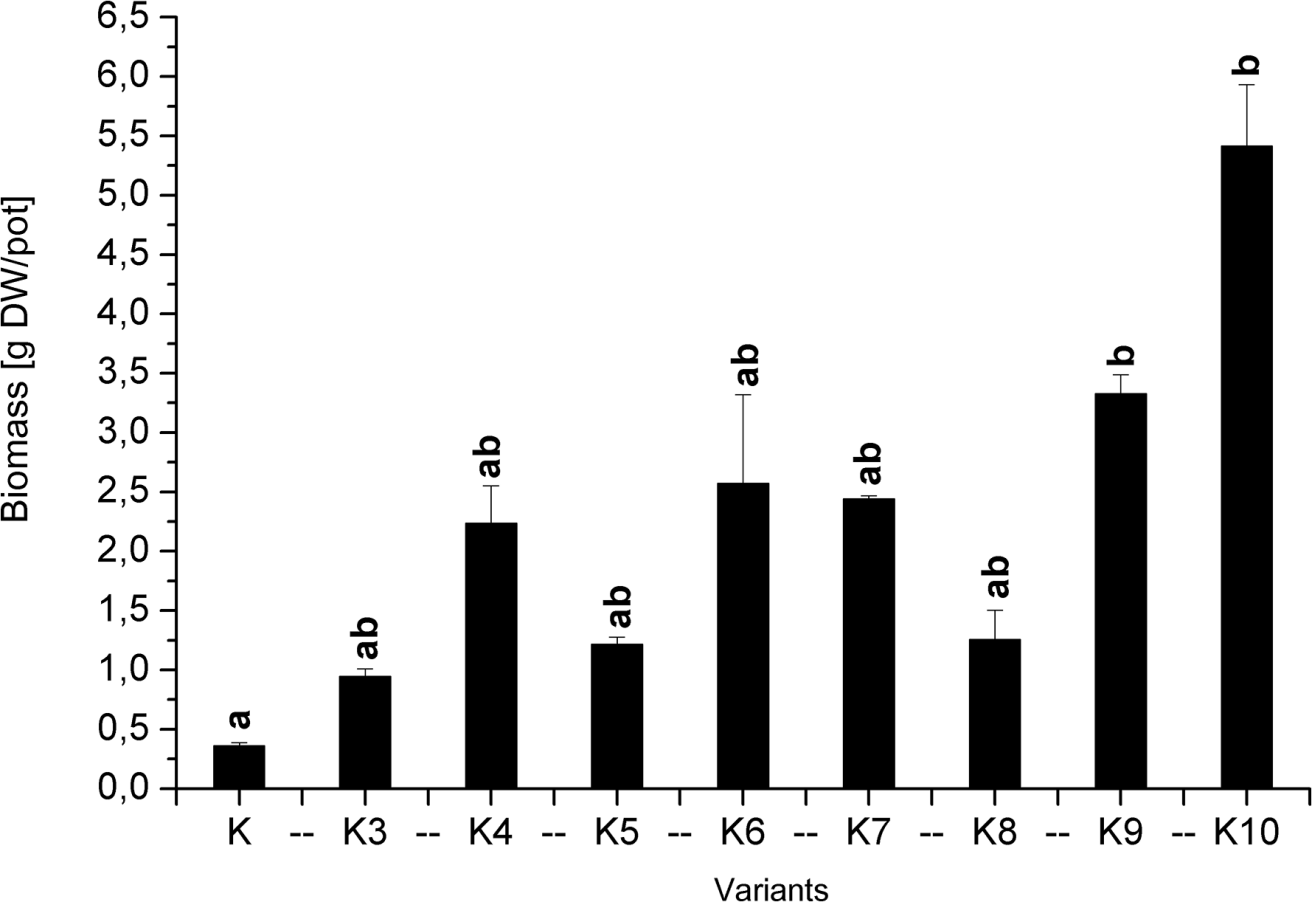
Plant biomass (shoots).

In a study of biomass growth (Fig 2), the highest yield (5.5 g d.w.) was obtained when both potassium phosphate and sewage was used and when superphosphate and sewage sludge application was combined (2.5 g d.w./pot). However, the use of only sewage sludge at 3% d.w. provided results that were similar (2.25 g d.w./pot) to those of the K6 and K7 combinations (2.25–2.50 g d.w./pot) with inorganic fertilisers. The treatment in which the soil was only limed (K) resulted in a very low shoots yield of 0.25 g d.w./pot. Moreover, the control pots provided only shoots biomass, because the roots dried up and thus not enough root biomass was collected.

**Fig 3:**
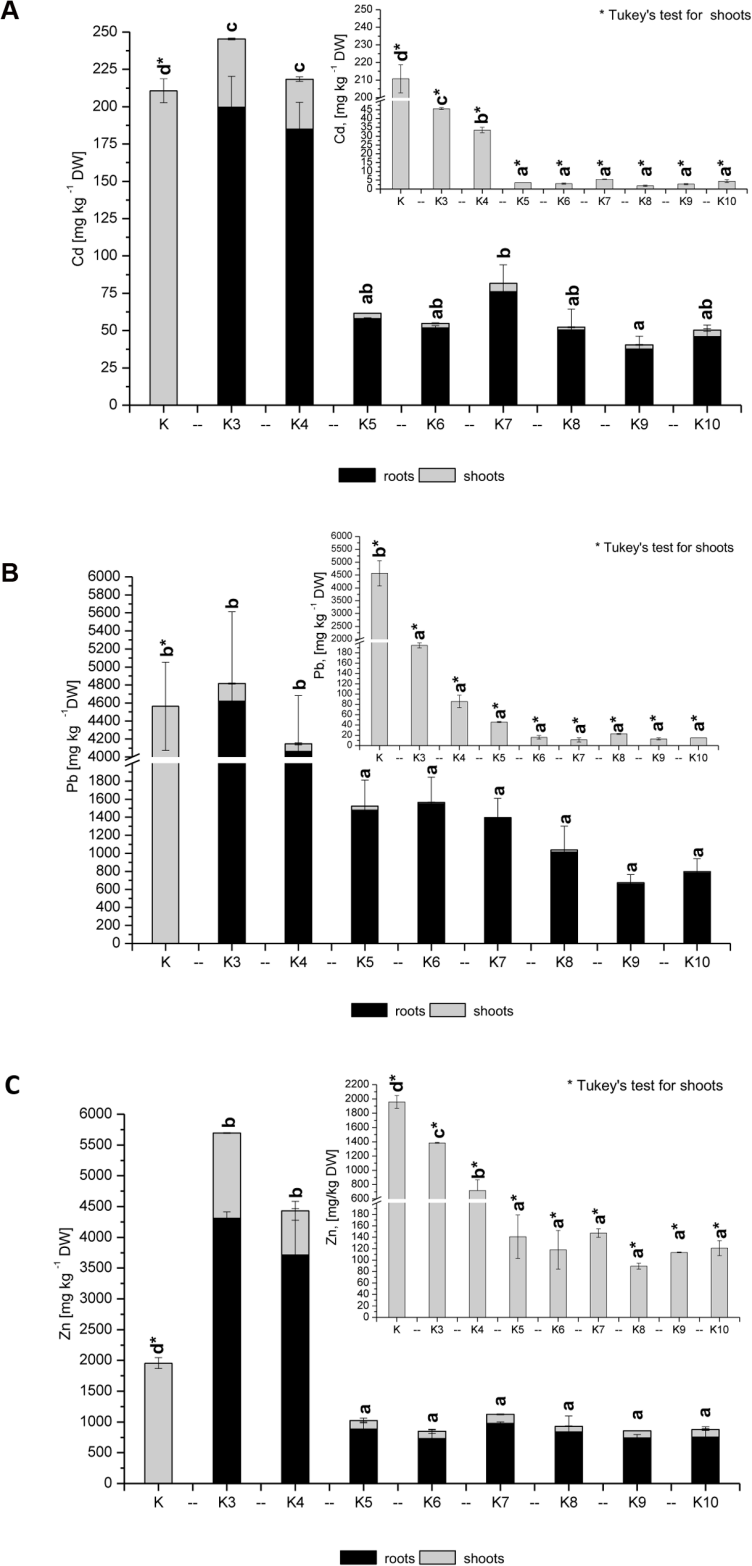
Changes in the Cd (A), Pb (B) and Zn (C) contents in the biomass [mg kg ^-1^ DW]; the control only shows shoots data (roots dried up and thus not enough root biomass was collected).

In a study considering all investigated metals, a very large decrease in the metal concentrations in the biomass was achieved after the addition of inorganic fertilisers (K5-K10) (Fig 3). However, the best results were obtained for variations in the combined applications of sewage sludge and inorganic additives. All metals were mainly accumulated in the roots.

For Cd, a decrease in the metal concentrations in the aboveground biomass was achieved (from 210 mg kg^-1^ in the control to 45–5 mg kg^-1^ for treated pots). In the roots of variants K3 and K4, the Cd concentrations were 190–200 mg kg^-1^ compared to the other combinations (K5-K10) with 75–30 mg kg^-1^. Generally, Pb accumulated in the roots, with higher concentrations for K3 and K4 (4000–4600 mg kg^-1^) compared with K5-K10 (1400–600 mg kg^-1^). The Zn contents in the roots for K5-K10 were much lower (up to 1000 mg kg^-1^) compared with those of K3 and K4 (3700–4200 mg kg^-1^).

### The sequential extraction results

**Fig 4:**
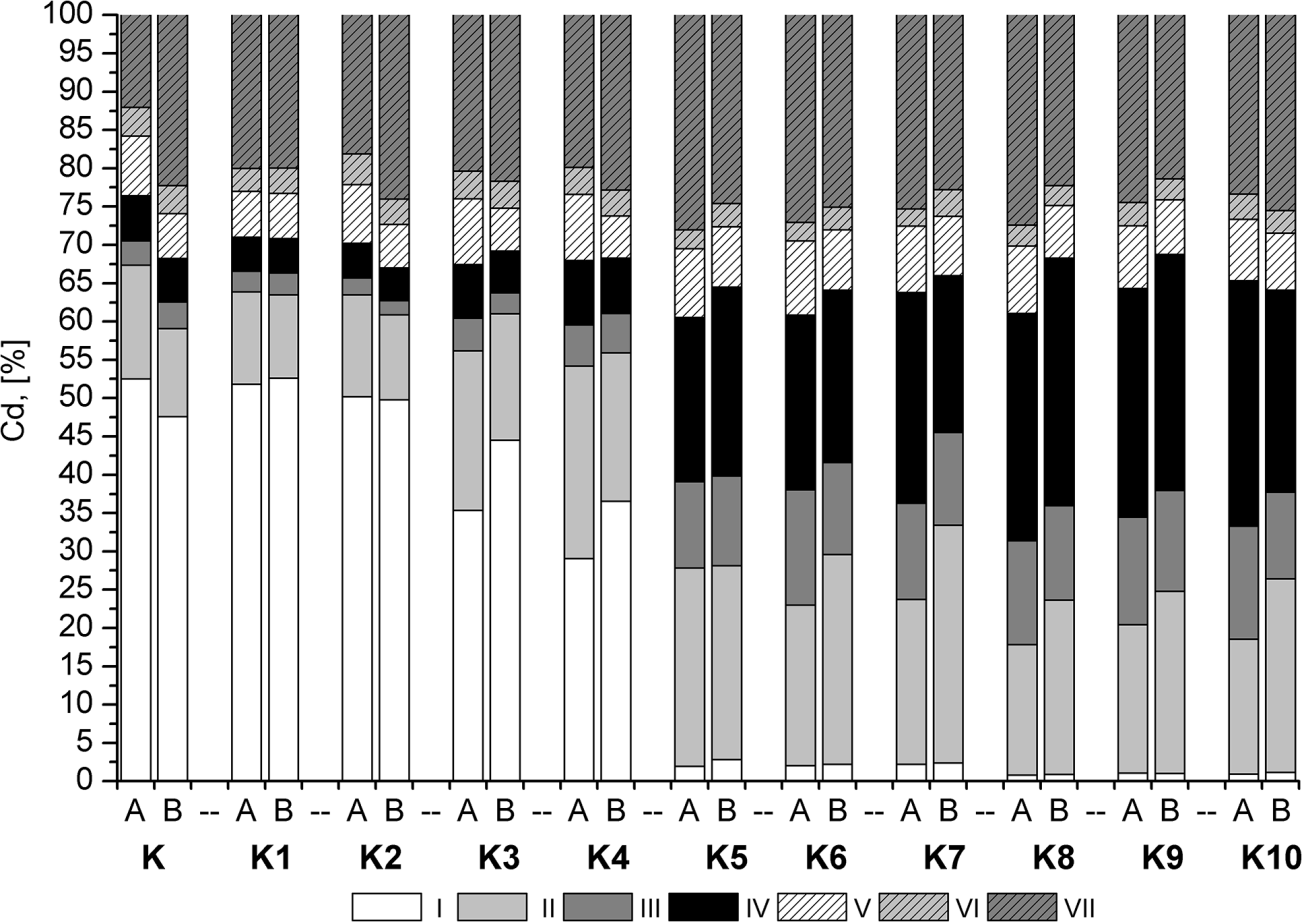
The results of the sequential extraction of Cd.

**Fig 5:**
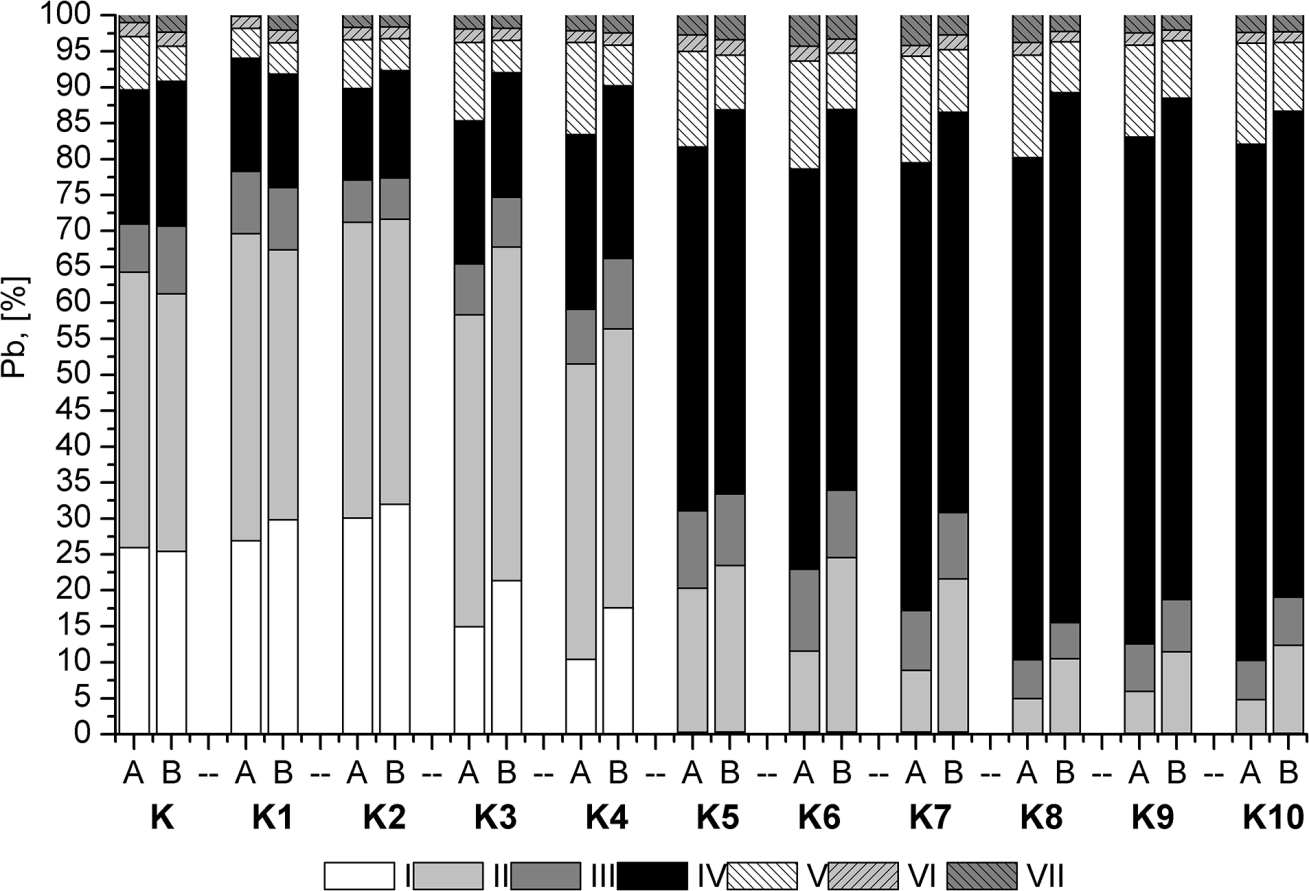
The results of the sequential extraction of Pb.

**Fig 6:**
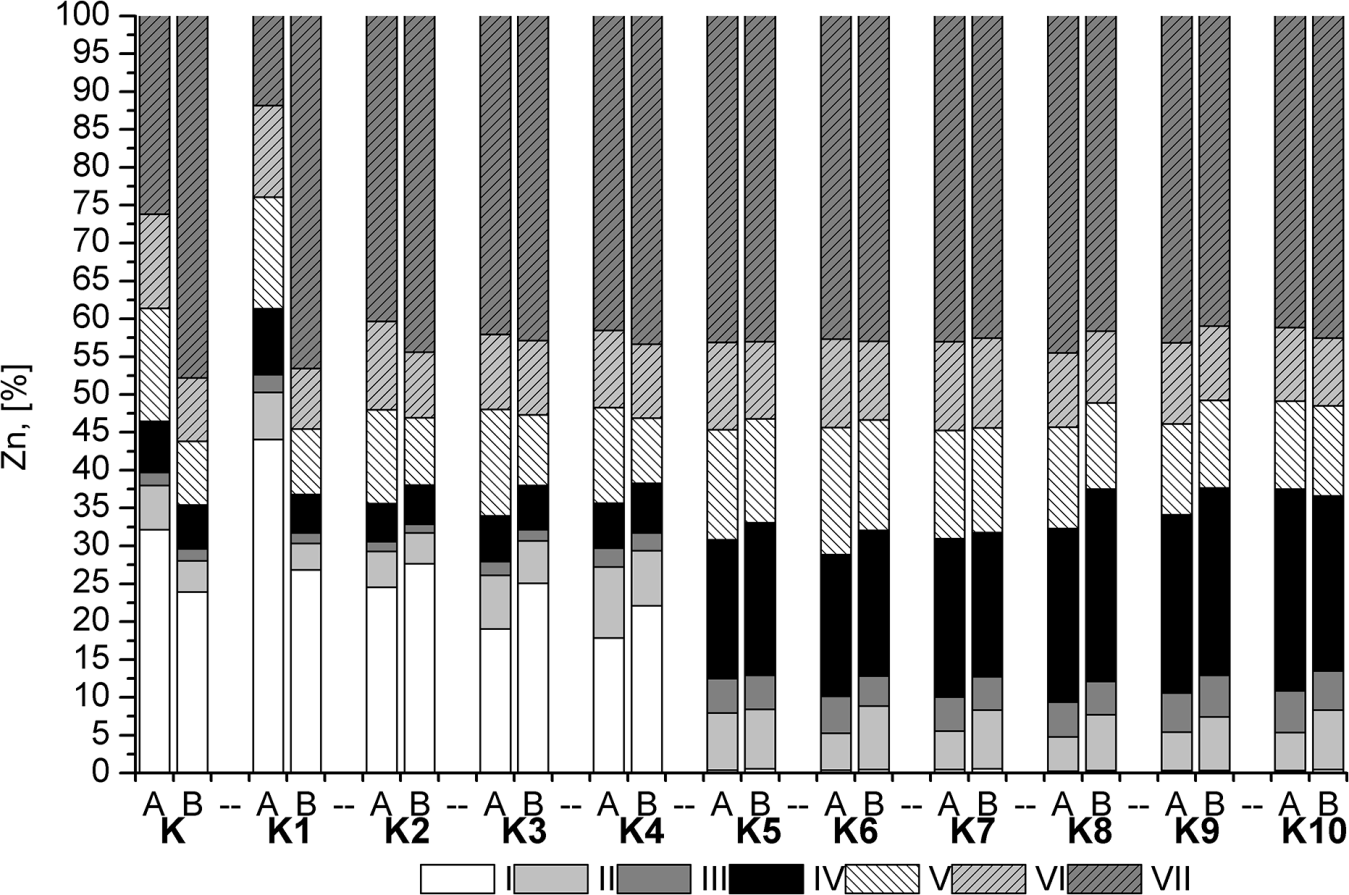
The results of the sequential extraction of Zn.

The Cd soluble fraction in the control samples was very high (50%), while the residual fraction accounted for a small percentage of the total Cd (Fig 4). The addition of sewage sludge resulted in a slight decrease in the soluble fraction. The inorganic additive concentrations decreased in fraction I to a few percent, or to 1% in K8 and K9 to 1%. In addition, Cd passed from fraction I to fractions II and IV. A large contribution of fraction III emerged, especially for the combinations of K8, K9, and K10 containing potassium phosphate. In a study of Pb, fractions I and II accounted for 30% of the total (Fig 5). The addition of sewage sludge resulted in a decrease in fraction I relative to fractions II and IV. In the combinations with inorganic additives, fraction I was not detected, and fraction II was reduced by half. Moreover, for a combination of K8, K9 and K10, this fraction decreased to a few percent. For Pb, a significant impact of plants on the decrease of fractions I and II was noted. For Zn, two dominant fractions were observed, I (30%) and VII (40%) (Fig 6). The application of sewage sludge resulted in a slight decrease in fraction I. Furthermore, this fraction was not detected after the application of inorganic amendments. An increase in fractions III and IV was observed.

## Discussion

Many factors affect the bioavailability, toxicity, and leachability of metals in the soil, however, study fractions are frequently omitted in research [12, 29]. The soil used in these experiments was characterised as containing bioavailable Cd, Zn and Pb. In addition, these metals were mainly associated with the readily soluble fraction (50% of the Cd, Zn 30%, Pb 25%). Similar results regarding the high bioavailability of the investigated metals in sandy soils were observed by Laszlo et al [30]. The high metal bioavailability in the tested soil resulted from the low pH, sorption capacity and organic matter content. Thus, the results from these soils were noted. In the literature, reports of properly growing plants in soils with much higher metal contents are given [31]. However, in this study, proper growth was not possible without the use of additives. One probable cause of this phenomenon could be the very high bioavailability of the metals (25–50% easily exchangeable), which is fairly rare in the literature [15], as well as the poor soil in which nothing was growing in the original environment. Such toxic metal concentrations in the bioavailable form, as reported in the literature [32], effectively inhibit plant growth in the very early stage. In this study, the combined use of sewage sludge and inorganic additives resulted in the highest significant decrease in the bioavailability of the investigated metals and finally resulted in decrease of investigated metals in plants biomass. To stabilise Pb, compounds containing phosphorus [33] are generally used to promote Pb precipitation to decrease its mobility and to promote ion exchange processes. In research regarding Pb, the highest immobilisation rates were obtained for variants of K8 (lime fertiliser and potassium phosphate 0.8%), K9 (lime fertiliser and potassium phosphate 0.8% with 1% sewage sludge), and K10 (lime fertiliser and potassium phosphate 0.8% with 3% sewage sludge). When combined with the use of inorganic additives, the bioavailability of Pb was severely limited. Huang et al. observed that no interaction effect occurred between the redox conditions or pH and the Pb fractions [34]. A decrease in Pb fractions (I, II) were noted in the presence of plants, which likely affected the rhizosphere acidification and increased the precipitation of P and Pb compounds. According to other authors, the bioavailability of Pb can be reduced by 90% [3, 33]. The tested sewage sludge variants and sewage sludge and inorganic amendments combinations and all inorganic (P based) amendments yielded similar, satisfactory results. This finding resulted from the dominant mechanisms of Zn complexation with organic matter and its role in cations exchange [3, 35]. Cd immobilisation was the highest for combined variants with the inorganic amendments: lime, triple superphosphate or potassium phosphate fertilizers and also sewage sludge combined with inorganic amendments. A large dose of sewage sludge amended solely did not cause the immobilisation effect and the mobility of metals was similar to control pots. The application of the inorganic additives decreased the Cd bioavailable (I) fraction to a few percent, or even to 1% in K8 and K9. In a study conducted by Basta and Sloan [36], a 53% decrease was obtained in Cd bioavailability after applying 180 g/kg of phosphate rock to the soil. In addition, the bioavailable fractions decreased by 90% following the addition of 10 g kg^-1^ of ammonium hydrogen phosphate. In this study, only a small degree of metal removal by plants was achieved and bioconcentration was mainly observed in the root zone. Soil additives during the phytostabilisation process were used to initiate plant growth and to improve the stabilisation / immobilisation of metals [2, 37].

The application of sewage sludge (K3, K4) did not increase the uptake of metals by plants to the aboveground parts of the plant compared to control without sewage sludge, which is in accordance with the results of studies conducted by Neuschutz [38]. Thus, the risk of biomagnification of heavy metals in ecosystems with the combined use of additives is very limited. Moreover, based on these results [30], the assumptions of the phytostabilisation process are met in this study. As confirmed by the research of Padmavathiamma and Li [39], fescue is widely used in phytoremediation due to its dense root system. Unfortunately, attempts to introduce this species in the contaminated areas without soil treatment were unsuccessful, which was confirmed by other researchers [3, 25]. This study also confirms, that the highest biomass can be achieved for sewage sludge and inorganics amendments treatment, compared to inorganic amendments treatment or only sewage sludge treatment. As confirmed in this study and according to the literature, sewage sludge improves the quality of the soil profile when its composition is appropriate and does not contain toxic substances that could negatively affect soil processes [37]. This finding is supported by the results of this research. The beneficial effects of compost from sewage sludge on soil properties were confirmed by other researchers [31]. The use of soil amendments, containing organic matter, for improving the soil quality in phytoremediation processes appears to be necessary.

Despite the favourable conditions for fescue growth (temperature, water), proper plant biomass was not obtained in the soils without additives. Studies have shown that Pb is mainly accumulated in the roots. This finding corresponds with results found in the literature [30], in which plants mainly accumulate Pb in their roots with limited transport to the aboveground biomass. Furthermore, the very mobile metals, Cd and Zn mainly accumulated in the roots and this confirms the proper selection of fescue grass for the phytostabilisation process. Moreover, this study confirms that the phytotoxicity of metals is mainly influenced by the form in which they occur (degree of bioavailability). Decreasing the bioavailability of metals is an important element for ensuring plant growth and development in phytostabilisation activities [3].

## Conclusions

This study aimed to confirm that multi-element soil contamination should be considered in terms of the number of simultaneously occurring factors that affect the bioavailability and migration of metals. In the technical aspect, we should look for solutions that improve many physical and chemical soil parameters (pH, Eh, sorption capacity, C, N, P, effects of plants rhizosphere) if the immediate cause of soil pollution cannot be removed. This mainly concerns sandy soils with a low bonitation class like brownfields which generally indicates a poor plant cover development. Contaminated soils that are devoid of vegetation are susceptible to erosion and leaching, which may lead to the subsequent transfer of pollution of the environment, should be subjected to the amended phytostabilisation treatments. In sandy soils that are highly contaminated with heavy metals, the initiation of secondary ecological succession processes, that involves pioneering vegetation and the development of plants from the next level of ecological succession, can be accomplished with the application of soil amendments. The fertilising effect of sewage sludge from the food industry is mainly a substantial improvement in the sorption capacity of the soil, as well as the enrichment of N, P and organic matter. For this reason, the use of a suggested technique of assisted methods of phytostabilisation, based on fescue grass, inorganic phosphate fertilisers and sewage sludge is justified.

The most preferred immobilisation results of the investigated metals (Cd, Pb, Zn), which was confirmed by decreasing both the bioavailable forms of metals in the soil and the total content in the plant biomass, were obtained for the combined application of triple superphosphate or potassium phosphate fertilisers and sewage sludge, compared to other variants. The sequential extraction of metals has confirmed that the most important impact on the Zn, Cd and Pb fractions has combined use investigated amendments. Thus, for the phytostabilisation of metals contaminated, sandy soils, the recommended, practical and promising technique is the use of sewage sludge from the food industry, potassium phosphate (PP) and lime (AML) at doses: 0.5% AML + PP 0.8% +1% sewage sludge, or 0.5% AML + PP 0.8% +3% sewage sludge. For practical use the recommended doses are in Mg hectare^-1^: 5.6 AML + PP 9 +11 sewage sludge, or 5.6 AML + PP 9 +33 sewage sludge.
